# Effect of Mirror Therapy for Patients With Shoulder Pain: A Scoping Review

**DOI:** 10.7759/cureus.95990

**Published:** 2025-11-03

**Authors:** Masaki Karasuyama, Yasuaki Tanaka, Daichi Miyazaki, Takashi Tsuruta, Junichi Kawakami

**Affiliations:** 1 Department of Rehabilitation, Minamikawa Orthopedic Hospital, Fukuoka, JPN; 2 Department of Rehabilitation, Juko Memorial Nagasaki Hospital, Nagasaki, JPN; 3 Department of Rehabilitation, Kugimiya Orthopedic and Rehabilitation Clinic, Oita, JPN; 4 Department of Anatomy, The Nippon Dental University School of Life Dentistry at Niigata, Niigata, JPN

**Keywords:** education, mirror therapy, rehabilitation, scoping review, shoulder pain

## Abstract

Mirror therapy (MT) is a noninvasive intervention that uses visual feedback to induce cortical reorganization and was initially applied to reduce phantom pain. More recently, MT has been adopted for upper limb rehabilitation, including shoulder disorders. While individual studies have suggested potential benefits of MT for pain and range of motion (ROM), no review has specifically focused on its use in patients with shoulder pain. This scoping review aimed to map the existing literature on MT in this population and identify knowledge gaps to guide future research. Searches were conducted in the Cochrane Controlled Register of Trials (CENTRAL), MEDLINE, and Scopus databases to identify studies published up to September 2024. To capture the most recent evidence, an additional search was performed in September 2025 to include studies released between September 2024 and September 2025. Study selection was independently carried out by two reviewers according to predefined eligibility criteria. Data were extracted on study characteristics, participant demographics, and outcomes related to the use of MT for musculoskeletal shoulder pain. The review process adhered to the Preferred Reporting Items for Systematic Reviews and Meta-Analyses extension for Scoping Reviews (PRISMA-ScR) guidelines. Of the 127 records identified, three studies met the inclusion criteria: two randomized controlled trials and one case series. Two studies investigated patients with adhesive capsulitis, and one study included individuals with nonspecific shoulder pain. Intervention protocols varied in frequency and duration, with session lengths ranging from three to 20 minutes and follow-up periods from one day to 10 weeks. Pain and ROM were the most commonly assessed outcomes, whereas functional, psychological, and quality-of-life measures were inconsistently reported. MT interventions generally involved active flexion, abduction, internal rotation, and external rotation of the unaffected limb viewed in a mirror. Reported findings suggest short-term improvements in pain and ROM; however, evidence for superiority over other interventions remains inconclusive. This scoping review highlights the emerging evidence on MT for patients with shoulder pain, particularly those with adhesive capsulitis. MT has been investigated primarily for its potential to improve pain and ROM, with functional outcomes assessed in only a limited number of studies. However, psychological and quality-of-life measures have rarely been evaluated, and heterogeneity in patient populations, intervention protocols, and outcome assessments limits comparability across studies. Future high-quality randomized controlled trials with standardized protocols and comprehensive outcome measures are needed to clarify the clinical effectiveness of MT and to define its role in rehabilitation practice.

## Introduction and background

Chronic shoulder pain is a common musculoskeletal complaint [1]. Although many cases can be treated effectively, a substantial proportion of patients continue to experience persistent pain even in the absence of a clear structural cause [2]. Psychological and psychosocial factors have been shown to affect both the onset and persistence of musculoskeletal pain [3]. Central sensitization, characterized as an exaggerated response of the central nervous system to nociceptive input, has been reported in 11-24% of patients with shoulder disorders [4]. These mechanisms may explain why chronic pain often persists despite conventional treatments [5-7].

Mirror therapy (MT) is a noninvasive intervention based on visual feedback that induces cortical reorganization and promotes neuroplastic changes without requiring active movement of the affected limb [8]. Originally introduced by Ramachandran to reduce phantom limb pain in amputees, MT uses the reflection of the unaffected limb in a mirror to create the illusion of pain-free movement of the affected side [9]. Since then, MT has been widely applied to upper limb rehabilitation. A Cochrane review demonstrated that MT improved motor function, activities of daily living, and pain after stroke, supporting its role as a complementary treatment to conventional rehabilitation [10].

Recently, studies have begun to investigate the efficacy of MT for shoulder disorders. However, the available evidence remains scarce, and the overall effectiveness of MT in musculoskeletal shoulder pain is still unclear. Accordingly, this scoping review sought to provide a comprehensive overview of the existing literature on MT for shoulder pain, summarize the current state of evidence, and highlight areas that warrant further investigation.

## Review

Materials and methods

Protocol and Registration

This review was conducted in accordance with the Preferred Reporting Items for Systematic Reviews and Meta-Analyses extension for Scoping Reviews (PRISMA-ScR) guidelines [11,12]. We registered the study protocol on the Open Science Framework (https://osf.io/nbghr/).

Search Strategy

We searched the Cochrane Controlled Register of Trials (CENTRAL), MEDLINE, and Scopus databases for studies published before September 2024. Since approximately one year had passed since the initial search and to capture newly published data before finalizing the review, we conducted an updated search in September 2025, covering studies published between September 2024 and September 2025. Searches incorporated specific keywords and Medical Subject Headings (MeSH) terms such as "shoulder pain", "shoulder impingement syndrome", "rotator cuff", "frozen shoulder", "shoulder instability", "rotator cuff tendinopathy", "mirror reflection", "mirror visual feedback", "mirror therapy", "mirror box", "mirror feedback", "mirror training", and "mirror illusion". In addition, the reference lists of all eligible papers and their citing articles were screened manually to identify further relevant studies. The World Health Organization International Clinical Trials Registry Platform and ClinicalTrials.gov were also reviewed to locate ongoing trials. Title and abstract screening, followed by full-text assessment, was independently conducted by two reviewers. Any discrepancies were discussed and resolved by consensus, with the involvement of a third reviewer when necessary.

Eligibility Criteria

Eligible research focused on MT for adults (18 years or older) experiencing musculoskeletal shoulder pain. The analysis included all published randomized controlled trials (RCTs), crossover trials, cluster randomized trials, quasi-randomized trials, non-randomized trials, observational studies with controls, case reports, and case series, regardless of language or country. Conversely, studies involving animal or cadaveric specimens and those reporting outcomes in patients with conditions indirectly related to shoulder musculoskeletal disorders (patients undergoing post-stroke rehabilitation) were excluded.

Screening and Study Selection

Two reviewers independently evaluated the titles and abstracts of all retrieved studies using the Rayyan software (Rayyan Systems Inc., Cambridge, Massachusetts, United States). Their screening results were compared, and any discrepancies were discussed until agreement was achieved; when consensus could not be reached, a third reviewer provided adjudication. In addition, the reference lists of the included papers were manually checked to ensure that no relevant studies were missed.

Data Extraction

To systematically collect information from the included studies, we organized a data summary table containing the following variables: (1) authors, (2) publication year, (3) country of origin, (4) study design, (5) participant characteristics, (6) sample size, (7) mean age, (8) intervention details such as session duration and frequency, (9) outcome measures, and (10) follow-up period. Prior to finalizing the table, pilot data extraction was performed on several studies to verify the consistency of the process. One reviewer extracted all data, while a second reviewer cross-checked the entries for accuracy. Any discrepancies were resolved through review of the original reports.

Risk of Bias and Quality Assessment

A risk of bias or methodological quality assessment was not conducted, as the aim of this scoping review was to map and summarize the existing evidence.

Data Synthesis

The extracted data were narratively synthesized based on study characteristics, intervention protocols, and reported outcomes.

Results

Study Selection 

**Figure 1:**
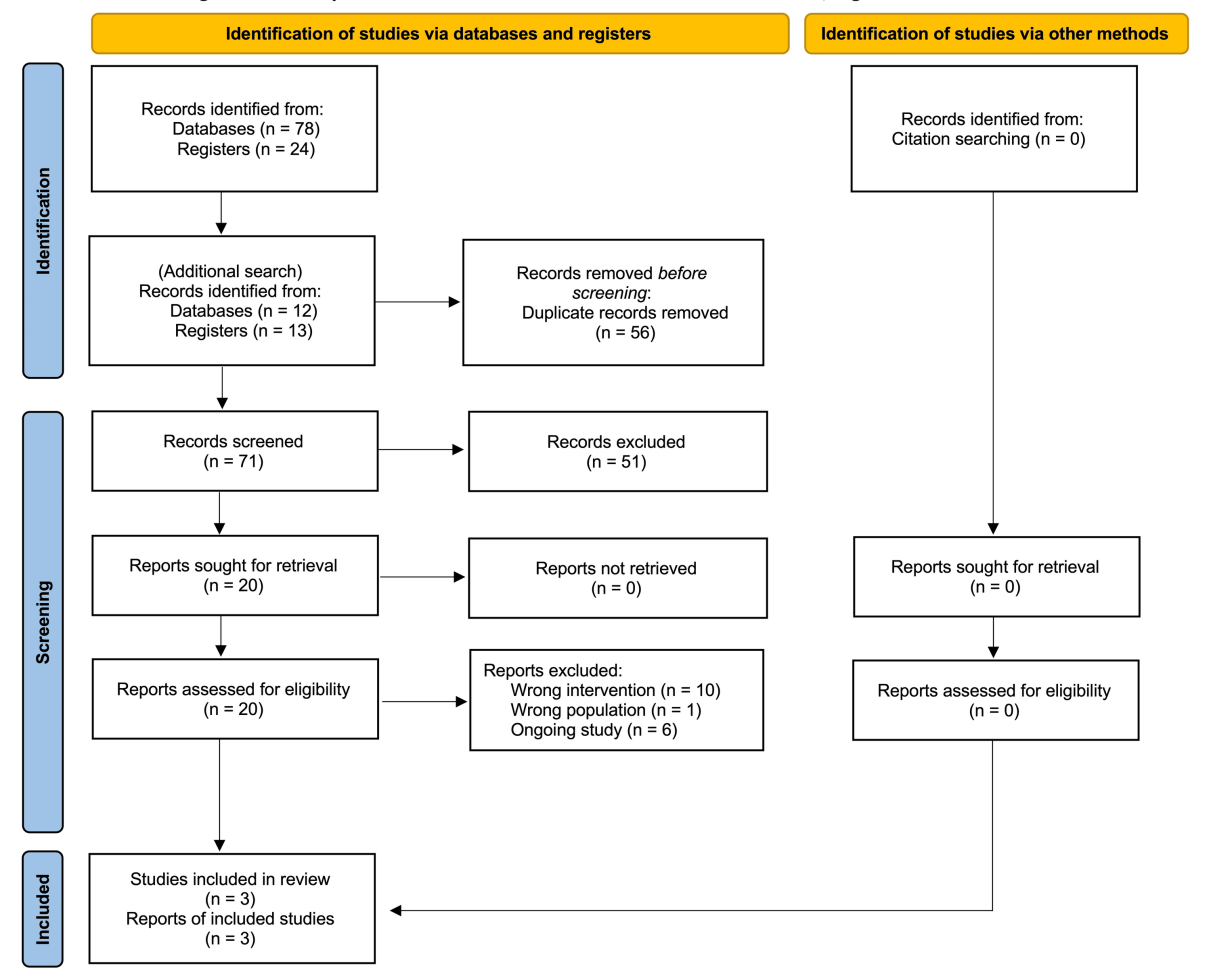
PRISMA flowchart of the articles included in the scoping review PRISMA: Preferred Reporting Items for Systematic Reviews and Meta-Analyses

Electronic searches identified 127 articles for screening. After removing duplicates, 71 articles were screened for eligibility. We screened 20 full-text articles and identified three studies [13-15] for inclusion in the scoping review. The overall selection process is shown in Figure 1, and the included studies comprised two RCTs [14,15] and one case series [13].

Description of Included Evidence

Among the three included studies, two [14,15] were conducted in Turkey, and one [13] was conducted in the United States. Of these, two [14,15] focused on frozen shoulder or adhesive capsulitis, whereas one [13] investigated patients with nonspecific shoulder pain. The characteristics and intervention details of the included studies are summarized in Table 1.

**Table 1:** Summary of mirror therapy interventions in the included studies

Author	Year	Country	Study design	Participant condition	Sample size	Tools (test group or subject)	Comparator	Number of sessions	Session length	Exercise content	Provider	Follow-up	Exercise description
Louw et al. [13]	2017	USA	Single-blind case series	Shoulder pain	69	Mirror	-	Single session	3 min	Flexion only	Physiotherapists	1 day	Active flexion of the unaffected shoulder in front of a mirror placed at the midline, creating the illusion of pain-free flexion of the affected shoulder
Başkaya et al. [14]	2018	Turkey	Randomized controlled trial	Adhesive capsulitis	Mirror therapy group: 15. Control group: 15	Standard physical therapy program + mirror (190×75 cm)	Standard physical therapy program + non-reflecting side of the mirror	10 sessions	20 min	Multiple (flexion, abduction, internal rotation, external rotation)	Physiotherapists	Not reported	Patients performed multiple shoulder movements of the unaffected side in front of a mirror positioned at the midline, producing the illusion of pain-free motion of the affected shoulder
Hekim et al. [15]	2023	Turkey	Randomized controlled trial	Frozen shoulder	Mirror therapy group: 12. Visual feedback group: 12. Control group: 12	Conventional physiotherapy + mirror	Conventional physiotherapy, conventional physiotherapy + visual feedback	15 sessions	20 min	Multiple (flexion, abduction, internal rotation, external rotation)	Physiotherapists	10 weeks	Similar shoulder movements performed with the unaffected side reflected in the mirror; additionally, a visual feedback group observed the affected arm directly during the same tasks

Regarding the outcome measures, three studies [13-15] used the pain score and range of motion (ROM); one study used the Pain Catastrophizing Scale [13], Tampa Scale of Kinesiophobia [13], University of California at Los Angeles (UCLA) Shoulder Score [14], 36-Item Short Form Health Survey (SF-36) [14], Shoulder Pain and Disability Index (SPADI) [15], Modified Constant-Murley Score [15], and Joint Position Sense Test [15]. However, the use of these measures varied across studies.

Intervention schedules varied substantially. Louw et al. examined the immediate effects of a single three-minute session [13]. Başkaya et al. implemented 10 sessions [14], and Hekim et al. delivered 15 sessions across five weeks (three times per week) [15]. Session length ranged from three minutes in Louw et al. to 20 minutes in Başkaya et al. and Hekim et al [13-15]. Exercise content also differed: Louw et al. [13] restricted the intervention to shoulder flexion, while Başkaya et al. [14] and Hekim et al. [15] included multiple movements (flexion, abduction, internal rotation, and external rotation). The number of repetitions and the time allocated per exercise were not consistently reported.

In all included studies [13-15], MT was administered by physiotherapists within supervised clinical or hospital-based rehabilitation programs. Follow-up periods varied across studies: Louw et al. [13] assessed outcomes immediately and one day post-intervention, and Başkaya et al. [14] did not provide detailed follow-up information, whereas Hekim et al. [15] conducted evaluations after completing a 10-week program.

Across all studies [13-15], MT consisted of active movements of the unaffected shoulder performed in front of a midline mirror, generating the illusion of pain-free motion of the affected shoulder. The task content varied from isolated flexion to multi-planar active movements.

Effectiveness of Intervention

Immediate effects (case series): Louw et al. [13] evaluated the immediate effects of a single MT session in patients with nonspecific shoulder pain. Statistically significant improvements were observed in pain, pain catastrophization, fear avoidance, and active shoulder flexion. However, most changes did not reach the minimal clinically important or detectable thresholds, with only a small proportion of patients meeting these criteria.

Mirror therapy vs. non-reflective mirror:* *Başkaya et al. [14] compared MT with non-reflective mirror exercises in patients with adhesive capsulitis. Both groups demonstrated improvements in pain and shoulder function; however, the MT group showed significantly greater reductions in pain and higher UCLA scores. Additionally, active and passive flexion and abduction ROM improved significantly more in the MT group. Quality-of-life assessment indicated superior gains in physical and emotional role functioning in the MT group compared with controls.

Mirror therapy vs. visual feedback vs. control: Hekim et al. [15] compared MT, visual feedback, and control interventions in patients with adhesive capsulitis. All groups improved after 10 weeks of physiotherapy. Visual feedback resulted in significantly greater reductions in pain compared with MT and control groups at both six and 10 weeks, while MT was not superior to controls. SPADI scores also improved significantly in the visual feedback group compared with controls, whereas no significant between-group differences were detected for ROM, proprioception, or Constant-Murley scores. Minor adverse effects included transient dizziness in three participants during MT, which resolved spontaneously.

Discussion

This is the first scoping review to examine the evidence on MT for patients with shoulder pain. The primary finding is the high degree of heterogeneity in patient populations, which makes comparison across studies difficult. This heterogeneity was also reflected in intervention protocols, session frequency, and outcome measures, limiting the ability to draw firm conclusions about the effectiveness of MT for shoulder pain. Nevertheless, all included studies reported improvements in pain and ROM, suggesting that MT may offer short-term benefits in certain clinical contexts.

The three studies included in this review differed in their patient populations [13-15]. One case series [13] investigated patients with shoulder pain, while the two RCTs [14,15] focused on adhesive capsulitis/frozen shoulder. Previous research suggests that pain sensitization occurs frequently in musculoskeletal shoulder disorders such as rotator cuff tears and impingement syndrome [3]. Therefore, MT may hold promise across a broader spectrum of shoulder conditions beyond frozen shoulder. However, the heterogeneity of study populations limits the generalizability of the current findings.

Pain and ROM were consistently reported as outcome measures across studies [16]. Additional assessments included psychological measures such as the Pain Catastrophizing Scale and Tampa Scale of Kinesiophobia, as well as functional scores (UCLA Shoulder Score, SPADI, and Modified Constant-Murley Score), quality of life (SF-36), and proprioception (Joint Position Sense Test). However, the use of these outcomes varied, and standardized assessment was lacking. This inconsistency limits comparability between studies and makes it difficult to establish the clinical significance of MT. Future studies should incorporate more comprehensive and consistent outcome domains, including quality of life and psychosocial factors, to better capture the multidimensional effects of MT.

Intervention protocols also varied considerably. Session duration ranged from three to 20 minutes, with follow-up periods ranging from one day to 10 weeks. In the case series by Louw et al. [13], a single session led to statistically significant improvements in pain, fear avoidance, and catastrophization, although most changes did not reach the minimal clinically important or detectable thresholds, indicating limited clinical relevance. Başkaya et al. [14] reported greater improvements in pain, ROM, and some quality-of-life domains in patients who received MT compared with controls. In contrast, Hekim et al. [15] found that although all groups improved after a 10-week physiotherapy program, MT was not superior to either the control or visual feedback groups. Interestingly, visual feedback produced greater pain reduction than MT, suggesting that the relative effectiveness of MT may depend on the underlying clinical characteristics of the disorder.

These findings provide insights into the potential mechanisms and applicability of MT. MT is proposed to act through visual-motor feedback, activation of mirror neuron systems, and modulation of central sensitization, mechanisms particularly relevant in conditions where altered pain processing predominates. This aligns with evidence presented in the introduction regarding the role of sensitization and psychological factors in chronic shoulder pain. At the same time, in disorders characterized primarily by mechanical stiffness, such as adhesive capsulitis, other approaches like visual feedback may prove more effective. Thus, MT may not be universally applicable across all shoulder disorders, and its relevance may depend on patient selection and clinical context.

Clinically, MT represents a low-cost, accessible, and noninvasive strategy that can be integrated into physiotherapy programs. However, given the limited number of studies, small sample sizes, and heterogeneity in design, interventions, outcomes, and methodological quality, no firm conclusions can be drawn regarding its effectiveness. Instead, this scoping review emphasizes that the current evidence base is sparse and heterogeneous. At present, MT appears promising for reducing pain and improving ROM in selected patient groups, but its role relative to other interventions remains to be clarified.

This review has several strengths and limitations. A strength is the use of a rigorous methodology with a priori protocol registration. However, only three studies were eligible, two of which involved patients with frozen shoulder, limiting generalizability. In addition, while one study assessed only short-term outcomes (immediate and one day) [13], another did not provide follow-up details [14], and the third assessed results at 10 weeks [15]; nevertheless, longer-term effects remain unexplored. Finally, heterogeneity in study design, intervention protocols, outcome measures, and methodological quality (e.g., lack of blinding) further limits confidence in the findings.

## Conclusions

This scoping review highlights the potential of MT in improving pain and ROM in patients with shoulder pain, particularly those with adhesive capsulitis. Pain and ROM were commonly assessed, whereas functional scores, psychological outcomes, and quality-of-life measures were reported less frequently. Future research should include high-quality RCTs with standardized protocols and comprehensive outcome measures to clarify the clinical effectiveness of MT and establish its role in rehabilitation practice.
